# Dynamic Protective Factors Relevant to Sexual Offending

**DOI:** 10.1007/s11920-024-01493-5

**Published:** 2024-02-24

**Authors:** David Thornton, Gwenda M. Willis, Sharon Kelley

**Affiliations:** 1 FAsTR LLC & SAARNA Inc, Ottawa, Canada; 2 University of Auckland, Auckland, New Zealand; 3 Sand Ridge Secure Treatment Center, Madison, Wisconsin USA

**Keywords:** Treatment planning, Case management, Dynamic risk, Protective factors

## Abstract

**Purpose of Review:**

Focusing on protective factors rather than risk factors potentially better aligns assessment with strengths-based treatment. We examine research into the assessment of protective factors to see whether it can play this role relative to sexual offending.

**Recent Findings:**

Structured asses sment of protective factors is well developed relative to violent offending but only recently studied relative to sexual offending. Nevertheless, multiple measures of protective factors have now been trialed with men who have committed sexual offenses and shown to predict reduced recidivism. Although research into individual scales is limited, overlapping content between scales suggests that protective factors aligning with constructs of Resilience, Adaptive Sexuality, and Prosocial Connection and Reward are all relevant to sexual offending.

**Summary:**

Protective factors relevant to sexual offending are sufficiently well identified that they can usefully be used for treatment need assessment, treatment planning during therapy, and case management. They can also make some contribution to risk assessment. The Structured Assessment of PROtective Factors against Sexual Offending (SAPROF-SO) is currently the most comprehensive measure of protective factors relevant to sexual offending.

## Introduction

Over the last two decades methods for the assessment of men who have committed sex offenses have been dominated by risk assessment tools. These can be divided into static actuarial instruments such as Static-99R, Static-2002R, and Risk Matrix 2000 [1, 2] which employ easily available statistical risk indicators, and measures of criminogenic needs such as STABLE-2007 [3] and the Violence Risk Scale – Sexual Offense version (VRS-SO; 4) which seek to identify psychologically meaningful risk factors that can be targeted in treatment. These nicely align with the risk-need-responsivity (RNR) model for effective interventions targeting reduced recidivism. The RNR model is well established with general correctional populations [5] and has been shown to apply to interventions that target risk for sexual recidivism [6]. Within RNR, static actuarial instruments can be used to prioritize individuals for treatment and to focus longer or more intense treatment on those presenting the greatest risk, thus conforming to the Risk Principle. Measures of criminogenic needs can be used to focus treatment efforts so that they prioritize making the changes that are most relevant to risk reduction, thus conforming to the Need Principle.

Over the last decade, there have been changes in the dominant approaches within treatment. The Good Lives Model provides a central framework for practice [e.g., 7•, 8••, 9] with criminogenic needs being understood as obstacles to attaining Primary Human Goods in a prosocial way while internal and external resources are strengths to be developed. Rather than solely addressing criminogenic needs, treatment influenced by this framework is increasingly oriented to either building on existing strengths or developing new strengths. This development in treatment practice promises treatment that is both more engaging and more individualized, but it has also led to a divergence between assessment practice and treatment practice: assessment focusing on deficits while treatment focuses on strengths.

Within the broader fields of correctional and forensic mental health treatment, an assessment practice has developed that could reconcile assessment and treatment. Several instruments now incorporate the assessment of what are variously labeled strengths or protective factors. This paper describes how the dynamic protective factors approach has begun to be extended to work with men who have committed sex offenses.

## What Are Dynamic Protective Factors?

Our motivation is to allow assessment to be complementary to strengths-based treatment approaches. To achieve this, dynamic protective factors need to be conceptualized in a way that makes them something that a person participating in treatment can aspire to manifest by deploying a pre-existing strength or by building up a strength that was absent or only weakly developed.

We count something as a protective factor if the presence of the factor is theoretically or empirically associated with a decrease in the rate at which a negative outcome occurs in a population for whom that outcome is a concern. Thus, a protective factor relevant to sexual offending would be something the presence of which among those with a history of sexual offending implied decreased sexual recidivism. A corresponding risk factor would be a factor the presence of which was associated with increased sexual recidivism. This conceptualization is similar to that of de Vogel et al. [10] and essentially identical to that of Willis et al. [11••]. Although there are some exceptions, most protective factors are at one end of a dimension that has a risk factor at the other end. Importantly, for something to be a protective factor, it must involve the positive presence of something that serves a protective function. A simple absence of risk factors is not sufficient. We do not make a distinction between strengths and protective factors: relevant strengths would meet our definition of protective factors. Despite the conceptual distinction between protective factors and risk factors, we see them as commonly interwoven so that, to some degree, the difference is that the same underlying dimension is being talked about in terms of what someone in treatment might strive for versus something they need to restrain. Nevertheless, protective factors can coexist with risk factors on the same dimension: for example, offence related sexual interest and prosocial sexual interest, or antisocial associates and prosocial associates. Furthermore, some potential protective factors do not have risk factors at the opposite pole. For example, where acute psychotic symptoms lead to an activation of dynamic risk factors [12], antipsychotic medication can operate as a protective factor. However, for individuals without such a major mental illness, not being medicated in this way is not a risk factor.

A related but different conceptualization was proposed in Loeber et al. [13]. They trichotomize variables that are empirically associated with offending, generally scoring them in the direction associated with higher risk. The trichotomization is based on distinguishing the 25% of the distribution at the negative (risky) end of the dimension, the middle 50% of the dimension, and the 25% of the distribution at the positive (less risky) end of the dimension. They label something as a risk factor if offending occurs more often for the negative end of the dimension than it does at the middle of the dimension while they label something as a promotive factor if offending occurs less often for the positive end of the factor than it does at the middle of the dimension. Under this scheme, dimensions can contain both risk and promotive factors, or just a risk factor, or just a promotive factor. This is a different conceptualization than the one adopted here. A promotive factor might or might not meet our definition of a protective factor depending on whether the positive end of the factor was defined by the presence of something. For example, they identify an absence of ADHD symptoms as promotive but that would not meet our definition of a protective factor. Similarly, if a moderate presence of something was all that was required for a protective effect, we would still describe it as a protective factor, while they would see it as solely a risk factor unless the positive end of the dimension was associated with lower recidivism than the middle of the dimension.

Work with risk factors has classified them in terms of whether they are statistical risk indicators with no intrinsic theoretical meaning versus psychologically meaningful risk factors [14] and among the latter a number of distinctions can be made in terms of their temporal properties. Long-term vulnerabilities [15, 16are relatively enduring risk factors that are liable to re-occur even if they are not currently present. This can be distinguished from how the person generally functions now. And that in turn can be distinguished from acute variation in the person’s functioning that may take place from day to day or hour to hour. In principle similar distinctions could be made for protective factors. Drawing on this framework, we use the phrase “dynamic protective factors” to refer to how psychologically meaningful protective factors generally manifest now, with “now” generally understood as a period of six to 12 months. We propose this temporal lens since briefer expression of protective factors may have less implication for stable future functioning while considering the expression of protective factors over decades would mean that they could not be aspired to in treatment. This means that we also are not concerned with what de Vogel et al. [10] refer to as static protective factors such as having had a secure attachment in childhood.

## Dynamic Protective Factors Relevant to Violent Offending

Wanamaker et al. [17] reviewed eight instruments that include what are labeled strengths or protective factors. Of these, the tool that focuses exclusively on protective factors and assesses them the most comprehensively is the SAPROF (The Structured Assessment of PROtective Factors for Violence; 18). Results with this tool are therefore the most relevant to our purposes. The scale includes three a priori sub-scales (Internal, Motivational, External), a total score, and a final protective judgment.

Burghart et al. [19••] reported a meta-analysis of results with this instrument. The mean ICC for the total score on the SAPROF based on 18 effect sizes was 0.80 indicating good interrater reliability. Interrater reliability for subscales was typically lower than for the total scale. A few studies examined interrater reliability for the Final Protective Judgment (an integration of item ratings using clinical judgement) and found the Final Protective Judgment was less reliable than summing items scores (mean ICC of .72 vs. .80). These results demonstrate that it is possible to assess protective factors with sufficient reliability for clinical practice.

Turning to the predictive value of assessing protective factors, Burghart et al. found the mean effect size (Cohen’s *d*) for prediction of decreased violent institutional misconduct from the SAPROF total score based on 14 effect sizes was .88. External protective factors which include external controls, lifestyle restrictions, and engagement with treatment services appeared less predictive than the internal factors such as self-control or empathy or motivational protective factors such as involvement in work, leisure, or positive life goals. Studies of recidivism after discharge showed a mean *d* of .63 based on 21 effect sizes for prediction of decreased violent recidivism from the SAPROF total scale. Again, prediction from external protective factors appeared to be weaker than from other kinds of protective factors.

Finally, Burghart et al. report that the incremental odds ratio of the SAPROF total score relative to violent recidivism controlling for scores on relevant risk assessment tools was examined in 11 effect sizes. Together, these studies indicated that SAPROF ratings add predictive information beyond that obtained from commonly used risk assessment tools.

An important limitation of this research is that although distinguishing external protective factors seems to have some value, the a priori subscales are not based on empirical research into item structure. This concern is reinforced by the findings of Abbiati et al. [20•] who tested the sub-scale structure using confirmatory factor analysis. This showed that the internal structure of the items did not correspond to the subscales. They followed up with an exploratory factor analysis which suggested four factors as follows: Resilience with highest loadings on Self-control and Coping; Reintegration with highest loadings on Leisure, Social Network, and Intimate Relationship; Treatability with highest loadings on Professional Care, Medication, and Motivation for Treatment; and Living Conditions with highest loadings on Living Circumstances and External Control.

## Dynamic Protective Factors Relevant to Sexual Offending

Although the SAPROF has primarily been studied in relation to future violence, some studies have examined its relation to sexual recidivism. Burghart et al. [19].

Another general measure of protective factors that has been tested in a sample of men who have committed sexual offenses is the Protective Strengths scale from the Inventory of Offender Risk, Needs, and Strengths (IORNS; [21]). The IORNS is a self-report measure which includes a 26-item Protective Strengths scale covering cognitive-behavioral regulation, anger regulation, education/training, and living situation. Miller [22] examined predictive validity of the IORNS in a sample of 89 adult males participating in sex offense specific treatment in Texas who had been out of prison for at least 6 years. Five of these 89 had sexually recidivated over this period. Miller reported an AUC relative to decreased sexual recidivism of .86 along with finding that the Protective Strengths scale’s predictive effect was incremental to the IORNS risk scales. This result is encouraging although the small sample size and very small number of recidivists means that there is a particular need for replication.

The VRS-SO is usually understood as a measure of dynamic risk; however, one of its scales, the Change scale meets our definition of a measure of protective factors. This scale is scored in relation to the individual’s long-term vulnerabilities and uses a modified stages of change rating so that higher scores indicate that the person has moved to a later stage of change. Olver has also described this as a movement from Offense Analog Behaviors (OABs) to Offense Replacement Behaviors (ORBs; [23]). Described more broadly, making change requires developing the motivation and skills to manage the individual’s long-term vulnerabilities so that healthy behavior can be deployed in place of dysfunctional behavior.

This seems to correspond quite well to Miller’s conception of protective strengths as including the ability to self-regulate. Olver et al. [4] reported a robust incremental association of Change with reduced sexual recidivism after controlling for both static and pre-treatment dynamic risk. This is based on four samples of adults treated in secure prison settings and a composite sample size of 913. The same pattern was shown for analyses of both five-year and ten-year sexual recidivism data. Interestingly, factor analyses indicated that while long-term vulnerabilities were organized in terms of three dimensions (Sexual Deviance, General Criminality, and Treatment Responsivity/Cognition), change was two dimensional, with one dimension involving the development of sexual self-management while the other Involved a developing ability to regulate antisociality.

The Structured Assessment of Protective Factors against Sexual Offending (SAPROF-SO; [11••]) is an adaptation of the SAPROF that was developed specifically for those with a history of sexual offending. Items from the SAPROF were rewritten, and additional items were added to better align the tool with the literature about protection relative to sexual offense recidivism (e.g., [24]). A 24-item pilot version was revised through initial studies into the 14-item SAPROF-SO Version 1. Willis et al. [11••] showed that excellent inter-rater reliability was possible with the pilot version (ICCs of around 0.9 in different samples) and that the overall protective factors score showed good construct validity. Kelley et al. [25] demonstrated how the instrument can be used in case management. Nolan et al. [26••] showed that high protection scores were associated with reduced sexual recidivism and that this effect was incremental to a well-established static actuarial risk assessment tool. Burghart et al. [19••] incorporated results for this instrument in their meta-analysis. Examination of the confidence intervals shown by these researchers indicated that the SAPROF-SO was more predictive of reduced sexual recidivism than the SAPROF. Willis et al. [27] reported that these results were retained by Version 1 despite the reduced number of items. Factor analysis of both institutional and community samples showed a three-factor structure: Resilience loaded strongly by Self-control, Coping, and Attitudes towards Rules and Regulations; Adaptive Sexuality loaded strongly by Prosocial Sexual Interests, Sexual Self-Regulation, and Prosocial Sexual Identity; and Prosocial Connection and Reward loaded strongly by Goal-Directed Living, Work, and Leisure.

Results from studies of these different scales should be viewed together as there is substantial conceptual overlap between them. This is illustrated in Table 1 which shows the higher loading items from corresponding factors or the authors’ summary of corresponding content.

**Table 1:** Overlap between different measures of protective factors

	SAPROF	SAPROF-SO	IORNS	VRS-SO
**Resilience**	Empathy Coping Self-control	Empathy Coping Self-control	Cognitive behavioral regulation Anger regulation	Regulation of antisocial traits
**Adaptive sexuality**		Sexual self-regulation Prosocial sexual interests and identity		Sexual self-management
**Prosocial connection and reward**	Leisure Network	Life-goals Work Leisure Network	Education Training	
**Treatability**	Professional care Medication Motivation for treatment	Sex offense specific treatment Therapeutic alliance Motivation for change		
**Living conditions**	Supervised living External control	Supervised living External control		

It is clear from Table 1 that the SAPROF-SO affords the most comprehensive coverage of the different factors found in these scales. The full set of items from the SAPROF-SO, organized by subscale, is shown in Table 2.

**Table 2:** SAPROF-SO version 1 items organized by subscale

**Resilience subscale**
Adaptive schema, Empathy, Coping Self-control, Attitudes towards rules and regulations
**Adaptive sexuality subscale**
Sexual self-regulation, Prosocial sexual interests Prosocial sexual identity, Intimate relationship
**Prosocial connection and reward subscale**
Goal-directed living, Work, Leisure activities Social network, Emotional connection to adults
**Professional risk management subscale**
Sexual offense-specific treatment, Therapeutic alliance, Motivation for managing risk Medication, Supervised living, External control

## Future Research

Research into dynamic protective factors relevant to sexual offending is in its early stages. There are significant opportunities for future research to refine our knowledge. While the SAPROF-SO is likely the most comprehensive measure of protective factors relevant to sexual offending it would clearly benefit both from more recidivism studies and from its construct validity being examined in additional populations. Relatedly, research relevant to sexual offending with all of these measures has been largely limited to adult males who have been convicted for a sexual offense. Studies with juveniles and females have not been attempted and instruments would need to be adapted (the SAPROF has an addition designed for females and an alternate version for juveniles but neither of these are specifically relevant to sexual offending). A more general issue is the application of instruments within groups with diverse identities. Hays [28••]’s ADDRESSING framework suggests consideration of Age and Generational differences, Disabilities, Religion, Ethnic and Racial identity, Social Class, Sexual Orientation, Indigenous Heritage, National Origin, and Gender Identity. Research addressing this issue can take at least two forms. First, it can investigate whether protective factors take different forms in groups with different identities. Qualitative research may be particularly relevant here and the results would speak to how protective factors manifest in different cultural contexts. Second, quantitative research can examine whether the protective effect of a factor, or group of factors, is equally large in different groups.

Even within the groups for which these instruments have been studied, only the VRS-SO Change score has been examined in a way that allows statistic integration of protective factors with actuarial risk assessment instruments. Additional recidivism studies oriented to quantitative integration are required to enable scores on measures of protective factors to be used to revise actuarial risk assessment.

Another area for development is the degree to which measures can be understood theoretically. Where this is possible research with the instrument can contribute to theoretical development (allowing theories to be falsified or revised in empirically based ways). It also makes it easier to use the results in case formulations. Some attempt has been made to do this with the VRS-SO [16•], with the SAPROF-SO, and theoretical understandings of protective factors have been proposed [29]. More work on this issue is clearly required.

## Potential for Practice

Although work with protective factors is relatively recent, it is sufficiently well developed to contribute to several areas of practice. Here, we consider treatment need assessment, treatment planning during therapy, case management, and risk assessment. We draw particularly on ideas from de Vogel et al. [30] and from Kelley et al. [25].

### Treatment Need Assessment

Treatment need assessment refers to the kind of assessment that might occur either as part of an aid to sentencing evaluation or as an initial part of a complex treatment intervention. In either case, its purpose is typically to identify relevant treatment needs which if addressed might lead to reduced risk. This kind of assessment typically would also speak to other issues such as the appropriate intensity of treatment or particular responsivity issues that should be taken into account. Importantly, the product produced by a treatment needs assessment is typically a communication to other professionals designed to contribute to their decisions regarding the individual being assessed.

Structured assessment of protective factors can contribute to a treatment need assessment in two primary ways. First, it allows the identification of protective factors that are present to a significant degree and which it will be important to sustain. This may alert the professional receiving the assessment of the potential to do harm by inadvertently disrupting existing protective factors. Second, it affords identification of insufficiently developed protective factors that should be a focus of clinical services. De Vogel et al. [10, 29] refer to this as the identification of Keys and Goals.

### Treatment Planning During Therapy

This refers to the ongoing negotiation of what the individual chooses to work on with a clinician with whom they have, or are developing, a therapeutic alliance. Strengths-based treatment planning will normally involve discussing what the person’s priorities and values are, along with what they were striving for when they committed offenses, followed by a collaborative identification of ways they can make their lives more satisfying while avoiding offending, and strengths they could use or develop to assist in this. Using the language of the Good Lives Model, the first part of this involves identification of valued primary goods that were implicated in their offending while the second part involves creating prosocial Good Lives goals and determining what strengths can contribute to achieving these goals. These can be pre-existing strengths that are newly purposed for this task or strengths that need developing. Additionally, one kind of strength can be used to develop a different kind of protective factor. For example, strengths that are part of the broad Resilience factor can be used to develop a lifestyle that reduces exposure to events that trigger dynamic risk factors or circumstances that provide opportunities to offend. Resilience can also make it easier to cope with the frustrations that sticking to such a lifestyle entails. Thus, Resilience can be applied to help someone develop an aspect of Sexual Self-Regulation.

Structured assessment of protective factors can contribute to this kind of Treatment Planning by providing a check on whether the focus of treatment that has been collaboratively evolved actually includes the main protective factors that need to be sustained and developed. If they are included, then there are reasonable grounds for supposing that risk will be reduced. If not, then the clinical process risks departing from the RNR Need principle and not conforming to the dual aims of GLM derived interventions: risk reduction and the enhancement of wellbeing.

**Fig. 1:**
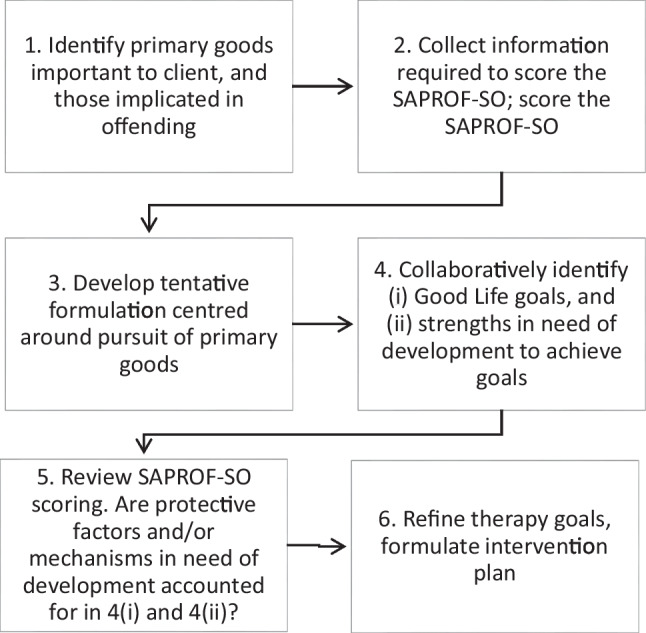
Therapy planning using the Good Lives Model and SAPROF-SO

Figure 1 illustrates the process described.

Where the treatment planning approach is less strengths based and simply focused on risk reduction, treatment goals may be framed in terms of controlling or suppressing risk factors. There is a place for this, but it can also be demotivating and potentially can set up various clinically unhelpful dynamics. One possibility is the development of an adversarial cycle in which the individual seeks to deny or conceal expressions of risk while the clinician seeks to identify risk factors by giving meaning to subtle aspects of the individual’s behavior. Another possibility is that the individual owns the risk factors that apply to them to such an extent that they become part of their identity. While the second possibility would make it easier for a therapeutic alliance to be developed, it is important to recall that modern understandings of the desistance process give a central place to the development of a prosocial identity, a view of the self that is not inherently deviant/criminal [31–34]. Framing what should be done to reduce risk as the development of protective factors can avoid some of these difficulties. Orienting to the development of protective factors as a way of reducing risk allows treatment goals to be formulated in a way that is more engaging and less stigmatizing. A focus on dynamic protective factors also gives the individual a greater sense of the future being in their control. A different kind of life becomes something they can choose.

### Case Management

By case management we mean a, typically multi-disciplinary, process in which decisions are made about things like the need for further treatment in the current setting, what kind of future setting would be most helpful, or whether services are no longer required. Case management might also speak to the form that service should take within a given setting. Structured assessment of protective factors can contribute to case management in a number of ways. Most straightforwardly it allows identification of how far protective factors have been developed in the current setting and affords discussion of which setting will make it easier to develop them further. A particular contribution is the theory of changing protections [30] which was also elaborated on in the context of the SAPROF-SO by Kelley et al. [25]. This asserts that potential protective factors can be divided into dynamically increasing factors and dynamically decreasing protective factors. The latter are professionally provided factors such as legal controls (supervision or a secure setting), a supervised lifestyle, or the provision of various kinds of treatment services, while the former are protective aspects of the individual or features of how they engage with their social environment that can continue after professional services are no longer being provided. Case management can then be understood as applying dynamically decreasing factors to the extent required to stabilize the individual but seeking to build up the dynamic increasing factors so that they can become the primary source of protection and so dynamic decreasing factors can be reduced in response.

Another role for the structured assessment of protective factors is for those individuals for whom professional services may be required indefinitely for them to live safely. Examples would be individuals with a traumatic brain injury whose compromised self-regulation easily leads to sexual or violent behavior or someone with major mental illness who becomes dangerous when their symptoms are more acute, and they decompensate. In the latter case, for instance, a mental health team may develop individualized behavior management plans that allow such individuals to be safely de-escalated and indicate how staff should interact with them to optimize their functioning. Kelley et al. [25] described how a structured assessment of protective factors should include determination of what processes within the current setting are required to sustain the individual’s current level of protective factors. This in turn can then be used to analyze a potential future environment to determine whether it can support this functioning.

### Risk Assessment

When the underlying research is better developed, it will be possible to make an actuarial adjustment of risk estimates derived from static actuarial instruments. At this time, we know that some adjustment of risk estimates based on the degree of protective factors is warranted but it is not clear how large this should be. In this state of partial knowledge the following strategy seems reasonable. Use a static actuarial risk estimate to characterize the pre-treatment level of risk, perhaps using the standardized risk levels [35]. Characterize the individuals’ long-term vulnerabilities. Factor analysis of instruments like the STABLE-2007 indicates that these cluster into two groups of related criminogenic needs, those involving antisocial traits and those involving offense-related sexual deviance [36]. It is then possible to use the SAPROF-SO to ask whether protective factors relevant to the person’s main long-term vulnerabilities have been developed. In doing this the Resilience group of protective factors should be considered relevant to antisocial traits while the Adaptive Sexuality group of protective factors is relevant to the sexual deviancy related long-term vulnerabilities. While we cannot yet precisely quantify the degree to which protective factors reduce sexual recidivism, we can be confident that they are associated with lower recidivism, so it is reasonable to use this kind of analysis to report whether someone has, for example, made risk relevant progress in treatment. If they have not made risk relevant progress in treatment, then their risk is likely well described by their score on the static actuarial instrument. If they have made this kind of progress, then their risk is likely materially lower.

## Conclusions

There is now compelling evidence for the role of protective factors relevant to violence. Evidence for the role of protective factors relevant to sexual offending is less developed. Nevertheless, when results with the SAPROF, the IORNS, the VRS-SO, and the SAPROF-SO, are considered together, cumulatively they provide substantive grounds for believing protective factors are also relevant to this kind of offending. Importantly, although there are significant differences between these instruments there is also considerable overlap. The SAPROF-SO provides the most comprehensive measure of protective factors relevant to sexual offending. The three primary factors it assesses (Resilience, Adaptive Sexuality, Prosocial Connection & Reward) are all assessed in at least one other instrument. Thus, Resilience is assessed by all the other three instruments, Prosocial Connection and Reward is assessed by the SAPROF and the IORNS. Adaptive Sexuality is assessed by the VRS-SO Change score.

Even in the current state of knowledge, measures such as the SAPROF in relation to violence and the SAPROF-SO in relation to sexual offending can make a useful contribution to practice, especially in the context of strengths-based treatment.
